# Informing decisions in light of parameter uncertainty – an economic evaluation of the adjuvanted recombinant herpes zoster vaccine in Sweden

**DOI:** 10.1007/s10198-025-01829-9

**Published:** 2025-10-14

**Authors:** Camilla Nystrand, Katarina Widgren, Shuang Hao, Emelie Heintz, Vibeke Sparring

**Affiliations:** 1 Department of Learning, Informatics, Management and Ethics, Karolinska Institute, Stockholm, Sweden; 2 Center for Health Economics, Informatics and Healthcare Research, Health Care Services Stockholm County (SLSO), Stockholm, Region Stockholm Sweden; 3 Department of Communicable Disease Control and Prevention, Stockholm, Region Stockholm Sweden; 4 Department of Medical Epidemiology and Biostatistics, Karolinska Institute, Stockholm, Sweden; 5 Department of Medicine, Solna, Karolinska Institute, Stockholm, Sweden

**Keywords:** Cost-effectiveness, Herpes zoster, Shingles, RZV, Adjuvanted recombinant zoster vaccine, Health economics

## Abstract

**Background:**

Many studies of the adjuvanted recombinant zoster vaccine (RZV) consider it cost-effective using efficacy estimates from randomized trials (RCTs). However, the effect magnitude differs between RCTs and observational studies, in addition to other input parameters that have shown to greatly impact cost-effectiveness. The aim of the current study is to assess the economic case of the RZV in Sweden and assess at which price the vaccine would be considered cost-effective.

**Methods:**

A decision-analytic model was used to estimate the health economic impact of introducing RZV in Sweden. Five-year age-cohorts were modelled between ages 65 to 100+, comparing the cost-effectiveness of two-dose RZV to no vaccination from a health care perspective, using efficacy data from RCTs and observational estimates. The model was run over a lifetime time horizon with quality adjusted life years (QALYs) as the outcome. Multiple one-way and probabilistic sensitivity analyses were conducted to analyze the impact of parameter uncertainty.

**Results:**

At a willingness-to-pay of 80,000 Euro per QALY, the RZV was cost-effective across cohorts at a price per dose of 80 to 105 Euro in basecase analyses, in contrast to the current market price at 176 Euro. However, due to parameter uncertainty, the price per dose at which the RZV may be considered cost-effective varies between as high as the current market price to less than 10% of that price, depending on which input variables are used.

**Conclusion:**

The price at which the RZV would be considered cost-effective varies greatly, highlighting the need to explore and consider parameter uncertainty in both analyses and procurement negotiations.

**Supplementary Information:**

The online version contains supplementary material available at 10.1007/s10198-025-01829-9.

## Background

The varicella-zoster virus is highly infectious, causing varicella (chickenpox). Once the primary infection has resolved, the virus becomes latent and can reactivate later in life, causing herpes zoster (HZ). Given that more than 95% of individuals over 50 years old have prior exposure to varicella globally, most people are at risk of developing HZ [1]. The lifetime risk of developing HZ for individuals who live until the age of 85 is approximately 30% [2, 3]. HZ occurs in all age groups, but with an increasing incidence after the age of 50. The incidence of HZ in Sweden is approximately 300 per 100,000 person years [4].

HZ is a much-dreaded disease, which typically manifests with prodromal pain followed by a localized rash [5]. Complications are common, particularly post-herpetic neuralgia (PHN) which is defined as long-term pain [6]. Other complications include meningitis, cranial nerve palsy and herpes zoster ophthalmicus. Complications of HZ can persist for months or even years, significantly affecting patients’ health-related quality of life (HRQoL) [7]. In addition, the societal burden is impacted further by increased health care resource use, with approximately 43% of total health care costs falling on primary care [8].

Currently, there is only one vaccine available to prevent HZ in Sweden, the adjuvanted recombinant subunit vaccine (RZV; Shingrix). It is not included in the Swedish national vaccination program, but available at an out-of-pocket expense. Several clinical trials have shown high vaccine efficacy of RZV against HZ and PHN, with long duration of protection, at least 73% after 10 years [9–12]. Previously, a live attenuated vaccine (ZVL) was also available, however, the vaccine efficacy of the ZVL was lower, of shorter duration and contraindicated in immunocompromised individuals. In recent years, the ZVL has been withdrawn from several markets, including the Swedish.

Albeit the promising evidence for RZV, the results seen in clinical trials contrast the relatively lower effectiveness evident from register-based retrospective studies [13, 14]. By comparison, the vaccine efficacy of individuals 50 years and older after one year was 97.6% (95% CI 90.9–99.8%) in clinical trials [11], while 85.5% (95% CI, 83.5–87.3%) using observational data [14]. Depending on which data are being used to inform policy making, it may have an impact on recommendations.

Previous economic evaluations have used vaccine efficacy estimations based on evidence from clinical trials [15]. A large majority of studies have concluded that the RZV is cost-effective, with varying incremental cost-effectiveness ratios (ICERs) associated with the waning of effects, reductions in HRQoL related to HZ and PHN, as well as the market price of the vaccine [15]. Using various willingness-to-pay thresholds, European cost-effectiveness evaluations have shown that the price per dose must be around 55–135 Euro for the RZV to be considered cost-effective [16–18]. In light of the lack of published studies assessing the cost-effectiveness of the RZV in Sweden, the aim of this study has been to assess the health economic impact of introducing RZV as a routine vaccination to the elderly in the Swedish context. Further, the study aims to assess at which price the vaccine would be considered cost-effective with regards to underlying parameter uncertainty and addresses the discrepancies in vaccine effectiveness between data from clinical trials and real-world registries.

## Methods

### Disease model and cost-effectiveness framework

**Fig. 1:**
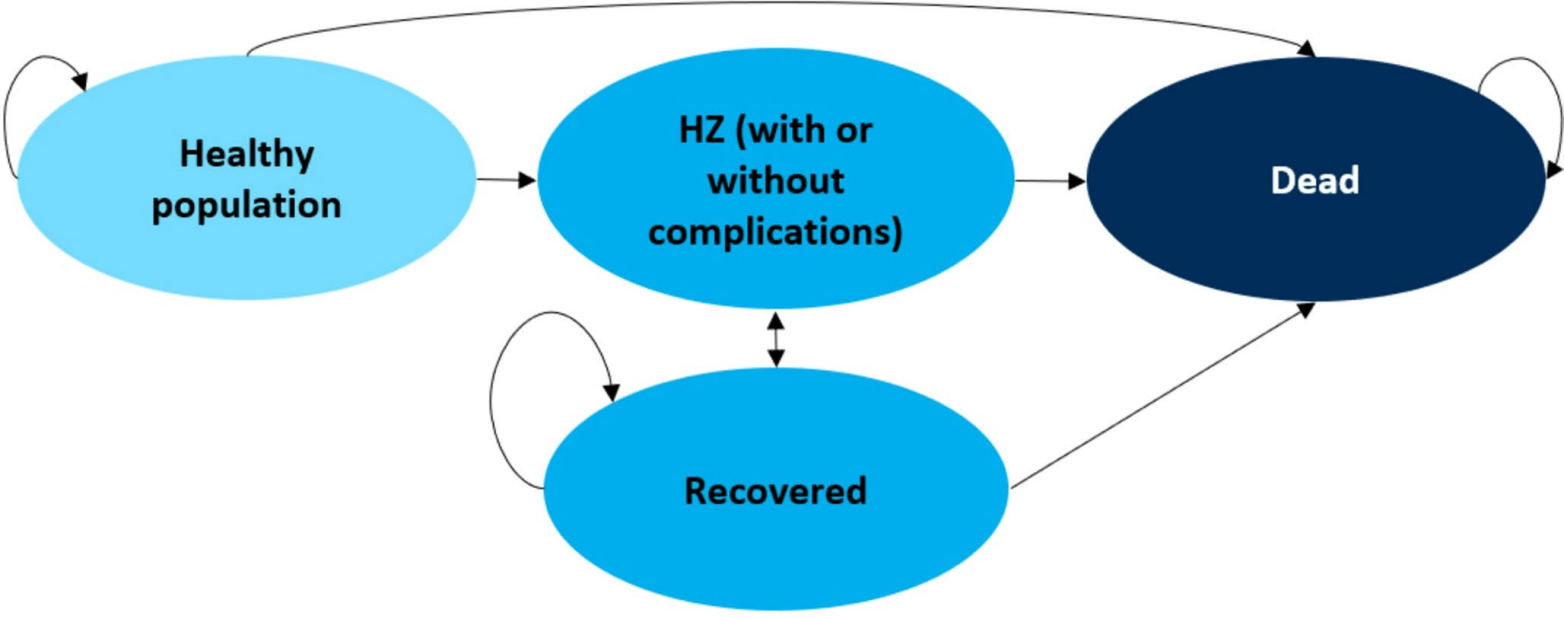
Conceptual model

A Markov decision-analytic model (Fig. 1) was developed and run in Excel to estimate the annual age- and- gender specific number of HZ cases in Sweden, with the corresponding treatment costs and HRQoL impact. The HZ state includes three tunnel states, depending on if individuals have HZ without complications, PHN or other HZ complications. The model simulated a cohort starting at the age of 65, given that the leap in incidence of HZ is from that age, the general recommendation of the zoster vaccine in Europe is from the age of 65, and most vaccination recommendations targeting elderly in Sweden start at the age of 65 [4]. Eight 5-year age-cohorts of individuals aged 65 to 100 + were projected in the model over a lifetime horizon, until 105 years of age. The time horizon is due to the long-term vaccine efficacy as well as the fact that HZ can reoccur. In the incremental analysis, a comparison was made between (1) 2-dose vaccination with RZV and (2) no vaccination. Annual cycles were used to model the population, where each age-cohort could remain healthy, develop HZ with or without complications, recover from HZ or die. The HZ state included additional health care costs and a reduction in HRQoL. Model structure, input and outcome variables were validated in comparison with similar studies and by clinical experts. A healthcare perspective was chosen, including costs related to the healthcare system and out-of-pocket expenses to individuals, as the starting age of the model is 65 and little productivity loss is expected. Costs and health outcomes after the first year were discounted at an annual rate of 3% in the base case analysis, as recommended in Swedish guidelines, and with other rates tested in sensitivity analyses [19].

### Model inputs

Input parameters are summarized in Table 1. Data specific for Sweden were used when available.

**Table 1:** Characteristics and source of input parameters used in the health economic model

Parameter	Estimate mean (standard error)			Uncertainty distribution	Source
*Demography* ^a^					
Cohort size (no.)	Female	Male		Fixed	[1]
65–69 years	272 568	267 652			
70–74 years	275 284	260 832			
75–79 years	251 189	231 607			
80–84 years	159 458	131 553			
85–90 years	100 350	66 971			
90–94 years	52 199	25 549			
95–99 years	15 643	5 239			
100 + years	2 226	436			
Probability of death from any cause	Female	Male		Fixed	[2]
65–69 years	0.010	0.008			
70–74 years	0.016	0.013			
75–79 years	0.027	0.022			
80–84 years	0.049	0.042			
85–90 years	0.093	0.080			
90–94 years	0.167	0.153			
95–99 years	0.251	0.315			
100 + years	0.353	0.434			
Incidence of HZ cases	Female	Male		Fixed	Stockholm regional healthcare database
65–69 years	0.0071	0.0051			
70–74 years	0.009	0.0068			
75–79 years	0.0119	0.0079			
80–84 years	0.0119	0.0092			
85–90 years	0.0117	0.0088			
90–94 years	0.0131	0.0089			
95–99 years	0.011	0.0042			
100 + years	0.0083	-			
Proportion of PHN (given HZ)	Both genders			Beta	Meta-analysis^b^
65–69 years	0.12 (0.022)				
70–74 years	0.15 (0.016)				
75–79 years	0.15 (0.016)				
80–84 years	0.15 (0.015)				
85 + years	0.16 (0.015)				
Proportion of HZ and PHN patients according to pain severity	HZ	PHN		Fixed	[3]
***< 70 years***					
No pain	0.65	NA			
Mild pain	0.24	NA			
Moderate pain	0.04	0.89			
Severe pain	0.08	0.11			
***> 70 years***					
No pain	0.45	NA			
Mild pain	0.41	NA			
Moderate pain	0.05	0.89			
Severe pain	0.09	0.11			
HRQoL general population	Female	Male		Beta	[4]
65–69 years	0.75 (0.01)	0.78 (0.012)			
70–79 years	0.66 (0.014)	0.76 (0.013)			
80 + years	0.57 (0.021)	0.68 (0.027)			
HRQoL weights HZ/PHN	Both genders			Beta	[5]
Mild pain (%)	0.91			β (81.27;8.08)	
Moderate pain (%)	0.71			β (153;62.55)	
Severe pain (%)	0.32			β (13.76;29.38)	
Number of healthcare visits	Both genders			Binomial (1000, mean/1000)	Stockholm regional healthcare database
	Primary care	Outpatient care	Inpatient care		
Zoster without complications B02.9	1.17	0.14	0.05		
Zoster with other nervous system involvement B02.2	1.38	0.27	0.14		
Zoster ocular disease B02.3^c^	0.87	1.82	0.17		
Disseminated zoster B02.7	1.00	0.22	0.15		
Zoster with other complications B02.8	1.25	0.21	0.11		
Mean no. of individuals prescribed medication	Antivirals	Analgesics			[6]
B02.9 (no complications)	62%	20%			
B02.0-B02.3 and B02.7-B02.8 (complications)	62%	50%			

^a^Data was stratified by age and gender in the model. Data shown in the table are divided by age-groups and presented for all genders

^b^Meta-analysis is presented in the Online Resource 1

^c^Due to low number of incidents B02.0 and B02.1 cases, healthcare visits were conservatively added to B02.3 due to it resulting in the lowest cost for primary, outpatient and inpatient care

### Demography

Data on population size and life expectancy in Sweden in December 2021 for age cohorts 65-years and older were retrieved from Statistics Sweden [20]. All-cause mortality data were obtained from the Swedish National Cause of Death register [21]. No HZ related mortality was assumed in the model due to low disease-specific mortality [8].

### Incidence of HZ and healthcare utilization

Age- and gender specific incidence rates and number of health care visits in year 2021 were obtained from the administrative healthcare data register VAL (Stockholm regional healthcare database). Although this was during the pandemic, incidence and utilization rates were compared to previous years and no major differences were detected based on face value. These rates were based on individual-level data, thus we were able to calculate both averages and variance in the data. In addition, aggregate data from other public registers in Sweden showed that the incidence and consultation rates in Stockholm were similar to the rates seen on a national level, which validates our individual-level data source used. The healthcare data register VAL contains pseudonymized individual level data regarding diagnoses, age, gender, hospitalizations, and other healthcare consultations within all publicly funded healthcare for all individuals in the region (1.76 million adult inhabitants). It includes information on primary care, outpatient specialized care and inpatient care. The ICD-10 code B02.9 as a primary or secondary diagnosis was used to identify incident HZ cases without complications, while only data from the individuals with HZ as a primary diagnosis were used to estimate healthcare utilization. To identify patients with HZ related complications, ICD-10 codes B02.0-B02.3 and B02.7-B02.8 were used. A wash-out period of one year was applied to ensure that all identified cases were new cases of HZ. The probability of recurring HZ was assumed to be the same as incident HZ, as our definition of incident cases includes both new and recurring cases and data on the true recurrence rate for the entire population show a wide range [22].

We categorized PHN as mild, moderate, or severe pain, and regardless of which level, it was defined as persisting for at least three months after rash onset. Different age-specific proportions (65–69, 70–74, 85 + years old) of PHN cases out of all HZ cases was estimated based on a meta-analysis of all relevant European studies on the topic, see Online Resource 1. The mean duration of HZ (1 month) and PHN (7, 10 and 13 months depending on severity) was retrieved from a UK study [23]. Healthcare utilization of individuals with PHN was identified through a Nordic register-based study [24].

Utilization of antivirals and analgesics related to HZ and PHN was obtained from Swedish register-based studies [4, 24]. Duration of use was based on clinical expert opinion.

### Incidence of stroke and healthcare utilization

The risk of stroke has in previous literature been seen to increase after HZ onset [25]. In the model, stroke was included in a sensitivity analysis, incorporating stroke-related healthcare utilization and quality of life decrements. The Swedish National Patient Register was used to retrieve age- and gender specific data on incidence rates of stroke [26]. An increased risk of stroke during the first year after HZ onset was assumed in the model [25]. Healthcare utilization related to stroke was based on estimates from Swedish register-based data [27]. All stroke related inputs are presented in Online Resource 1.

### Quality-adjusted life years

The decrease in quality of life related to HZ and PHN used in the base case analysis was obtained from van Hoek et al. [28]. The proportion of individuals with no pain, mild, moderate or severe pain [23] was multiplied by the HRQoL decrement due to HZ or PHN. The reduced HRQoL was estimated as a percentage of the baseline health state value for each pain state. The baseline health state values were based on the national age- and gender specific HRQoL estimates for the Swedish population [29], see Table 1. We assumed that individuals experienced mild, moderate, and severe pain for 7, 10 and 13 months respectively. Further, we assumed that individuals went back to their original (baseline) level of HRQoL (health state value) directly after the pain episode. Both the baseline health state value and changes in that value due to HZ and PHN were measured using EQ-5D-3L, with a majority of studies applying health state index values from the UK value set [30]. A further decrement in HRQoL related to the first year after stroke was applied in a sensitivity analysis [31], using the multiplicative method [32]. HRQoL decrements due to HZ, PHN and stroke were assumed to be constant for all ages and genders. In several sensitivity analyses, other HRQoL decrements related to HZ and PHN were applied, which have been used in previous European health economic evaluations of RZV. These were derived from a recent meta-analysis on the topic [15], and outlined in the Online Resource 1.

### Costs

All costs measured were in 2021 Swedish krona (SEK), converted to Euro (exchange rate 100 SEK = 10.15 Euro). Unit costs are presented in Table 2.

**Table 2:** Unit cost parameters used in the health economic model, 2021 Euro

Parameter	Cost per unit	Source
Vaccine administration	7.2	
Vaccine price per dose, basecase (RZV)	176.4	[7]
Primary care visit	182	[8]
Outpatient care visit		[9]
Zoster without complications (B02.9)	369	
Zoster encephalitis, zoster meningitis, zoster ocular disease (B02.0, B02.1, B02.3)	193	
Zoster with other nervous system involvement (B02.2)	613	
Disseminated zoster (B02.7)	393	
Zoster with other complications (B02.8)	384	
Inpatient care	5,180	[9]
Antivirals (per dose)^a^	0.72	[10]
Analgesics (per dose)^a^	0.18	[10]

^a^ Estimates based on the cost per dose of all brands/quantities available on the Swedish market for antivirals and analgesics

The direct healthcare costs were obtained from the Swedish National Cost per Patient registry for the year 2021, using data from Region Stockholm [33]. For reimbursement purposes, all publicly funded healthcare providers report individual level cost estimates based on ICD-10 codes. The cost for HZ without complications (B02.9) was age and gender specific. Due to the small number of patients for each specific HZ complication, the cost was assumed to be the same across ages and genders. As the registry does not contain any primary care costs for Stockholm, a regional price list was used to obtain information on the cost of a regular primary care visit [34].

For vaccine administration cost, we used the reimbursement cost for vaccination against seasonal influenza in Stockholm [35]. For the vaccine, we used the private sector cost per dose (176 Euro) which was varied throughout analyses [36].

Further, the cost for medication related to antivirals and analgesics was included as an average cost per HZ/PHN case. We assume antivirals are prescribed for a week in accordance with national guidelines, summing to 18.7 Euro, while analgesics are prescribed for the whole period of pain, ranging between 7 and 13 months, with a cost of 16.6 Euro per month. Costs per dose were obtained from the Swedish Dental and Pharmaceutical Benefits Agency [27].

### Vaccine coverage and compliance

Based on the current annual vaccination coverage of influenza vaccine in Stockholm, the coverage of the RZV was assumed to be 63% [37] in the base case analysis. We further assumed that 74.9% of individuals vaccinated with the first dose comply with the second dose [38]. Vaccine coverage and compliance to both doses were tested in sensitivity analyses.

### Vaccine efficacy and effectiveness

Two scenarios were used to estimate the cost-effectiveness of the RZV, using data from (I) RCTs and (II) observational studies. Both scenarios were compared to no vaccination, as the vaccine efficacy studies compared vaccination against no vaccination. In addition, the efficacy of the ZVL vaccine, which at that time had been available for about a decade in Sweden, wanes fast and was relatively expensive as an out-of-pocket payment compared to other vaccines [36, 39]. We thus assume a large majority of the population has no current protection against HZ from a previous vaccination. For the first scenario (I), interim results from two long-term follow-up RCTs were used as estimations for the efficacy of the RZV up until ten years post vaccination of two doses [40, 41]. In the RCTs, the vaccine efficacy was tested on individuals 50 years of age or older, who did not have an immunosuppressive condition. The efficacy at year one was 97.7% (95% CI 93.1–99.5%). An observational study measuring vaccine effectiveness was used for the effect after only one dose [13], which was applied only during the first year in the model due to waning uncertainty. The effect of the two-dose regimen post ten years was assumed to be waning with the average rate of the first ten years, which corresponds to a waning of 3.1% annually. In the second scenario (II), meta-analytic results based on two observational studies were used for the vaccine effectiveness one-year after the two-dose regimen [42], which was 79.2% (95% CI 57.6–89.7%). In the observational studies, the vaccine effectiveness was evaluated for a population aged 50 vs. 65 years and older, regardless of underlying health conditions or with immunocompromised individuals excluded. Post one-year, a 3.1% annual waning was applied, informed by the RCTs in the first scenario. The effectiveness after only one dose was applied the same way as in the first scenario. While these estimates have been used in the base case analysis, sensitivity analyses included a more conservative estimate with no protection against HZ post ten years. The vaccine effectiveness over time was applied to both first and recurrent HZ incidence. The vaccine efficacy for both scenarios over the modelling period is presented in Online Resource 1.

### Outcomes and cost-effectiveness analysis

The Markov model was used to estimate avoided HZ cases, QALY gains, avoidance of HZ related costs, and vaccination costs. Incremental QALYs and costs for the scenario I and II versus no vaccination was estimated to assess the cost-effectiveness of the RZV. A range of willingness-to-pay (WTP) thresholds per QALY gained was used to assess the probability of cost-effectiveness. The price of the vaccine was varied in the analyses to explore at what price the vaccine would be considered cost-effective at a WTP of approximately 80,000 Euro, which has been estimated to be the average accepted WTP for reimbursement of drugs for non-severe diseases in Sweden [43]. Results for the age cohort 65–69-year-olds are highlighted in the main manuscript, since this was considered a likely target-age for reimbursed vaccination in Sweden, while graphs also depict results for all age-cohorts. Results for combined age-groups of 65+, 70 + and 75 + years and older are presented in Online Resource 1. These combined results are relevant in the case of a catch-up program, covering vaccinations for the entire elderly population. For the combined age-groups, the incremental cost-effectiveness ratio (ICER) was estimated as the average ICER weighted by population size of each age-cohort.

### Sensitivity analyses

A probabilistic sensitivity analysis was run to account for variance of the input variables. In total, 1,000 Monte Carlo simulations were performed, by which we obtained the 2.5th and 97.5th percentile, allowing for a 95% credible interval of the results. In addition, several univariate sensitivity analyses were performed (Table 3), testing for robustness of results in relation to structural uncertainty (discount rate, starting age) and general assumptions (vaccine uptake, duration of vaccine protection). As five European economic evaluations of the RZV used different HRQoL decrements related to HZ and PHN, a parameter that has been identified as having a large impact on the results [15], these decrements were used in sensitivity analyses.

**Table 3:** Parameters and assumptions in the base case and one-way sensitivity analyses

Assumption in basecase analysis	Sensitivity analyses
3% discount on costs and effects	1. 0% discount on costs and effects 2. 5% discount on costs and effects
Vaccine uptake dose 1: 63%	1. 50% 2. 70%
Vaccine uptake dose 2: 74.9% of dose 1-recipients	1. 50% 2. 100%
Vaccine efficacy after two doses waning at a rate of 3.1% after 10 years	No protection from vaccine after 10 years
Only direct costs associated with HZ and complications	Including costs and QoL changes due to the increased risk of stroke
Vaccine efficacy equal over age-cohorts	Age specific vaccine efficacy, see Online Resource 1
Health state values for HZ and PHN based on van Hoek [11]	Same health state values for HZ and PHN as reported in the following studies (see Online Resource 1): 1. Pieters et al. [12] 2. van Oorschot et al. [13]/Curran et al. [14] 3. Ultsch et al. [15] 4. de Boer et al. [16]
Mean estimate of proportion of individuals with PHN from own meta-analysis^a^ (See Table 1)	1. Lowest confidence interval limit from meta-analysis (see Table S2 Online Resource 1) 2. Highest confidence interval limit from meta-analysis (see Table S2 Online Resource 1)
Unit costs as reported by various sources (see Table 2)	1. 40% lower unit costs 2. 40% higher unit costs

^a^See Online Resource 1

### Subgroup analyses

Subgroup analyses for males and females were performed and results are presented in the Online Resource 1.

## Results

### Base case analyses

Using data from 2021, the estimated total number of HZ cases in Sweden for individuals aged 65–69 years would amount to 85 000 cases over their remaining lifetime (total number of individuals 65–69 = 540,220, see Table 1). Vaccinating individuals with RZV, assuming the same coverage as for the annual seasonal influenza vaccination (63% of the population) and a 75% adherence to the two-dose regimen, would cost roughly 110 million Euros (at a price per dose of 176 Euro), including administration costs. It would avert an estimated 27 011 HZ episodes using data from clinical trials (scenario I), and 22 481 episodes using data from observational studies (scenario II). Similarly for PHN, about 30% of the cases would be averted by vaccination. The results are presented in Table 4.

**Table 4:** Number of cases, discounted QALYs, discounted costs and incremental cost-effectiveness (2021 Euro) related to the RZV (using efficacy data from clinical or observational studies) vs. no vaccination for individuals aged 65-69 years. Mean estimates and 95% credible intervals (CrI) are shown

Outcomes	RZV	No vaccination	Incremental difference^a^
Scenario (I) – efficacy data from clinical studies			
HZ cases	58 248	85 259	-27 011
PHN cases	10 371	15 034	-4 663
QALYs	5 326 231	5 325 650	581 (513 – 643)
Vaccination costs^b^	109 256 637		
Direct costs due to HZ and complications	629 605 783	653 738 766	-24 132 983 (-28 844 138 – -20 333 886)
Net cost			85 123 655 (80 412 499 – 88 922 752)
Incremental cost/QALY			147 110
Scenario (II) – efficacy data from observational studies			
HZ cases	62 782	85 263	-22 481
PHN cases	11 147	15 030	-3 883
QALYs	5 326 374	5 325 892	482 (423 – 545)
Vaccination costs^b^	109 256 637		
Direct costs due to HZ and complications	633 577 782	653 658 592	-20 080 810 (-24 065 087 – -16 972 949)
Net cost			89 175 827 (85 191 550 – 92 283 689)
Incremental cost/QALY			185 783

^a^Incremental differences can differ slightly from just subtracting numbers from the “no vaccination” and the RZV columns, due to probabilistic sampling

^b^Based on a vaccine price of 176 Euro per dose

By averting HZ and PHN cases, HRQoL of the affected individuals would remain at a higher level, while direct health care costs would be avoided. For scenario (I), using efficacy data from clinical trials, QALY gains would amount to 581 (95% CrI 513–643) over a lifetime (0.001 per person), while direct costs due to HZ and related complications were estimated to decrease by 24.1 (95% CrI 20.3–28.8) million Euro (45 Euro per person). In scenario (II), the amount of QALYs gained in the population were estimated to 482 (95% CrI 423–545) over a lifetime (0.001 per person), while direct costs were reduced by 20.1 (95% CrI 17-24.1) million Euro (37 Euro per person).

**Fig. 2:**
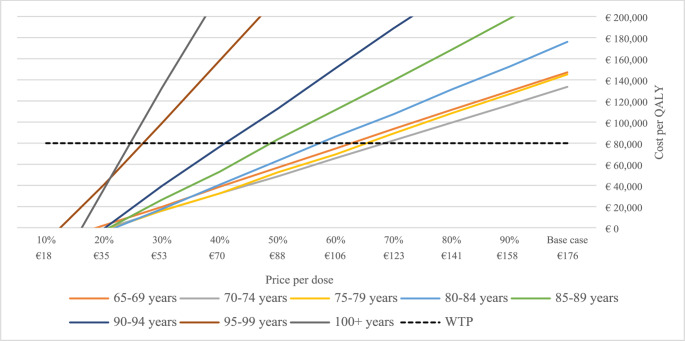
Cost per QALY at different prices of the vaccine for scenario (I) using clinical trials data

**Fig. 3:**
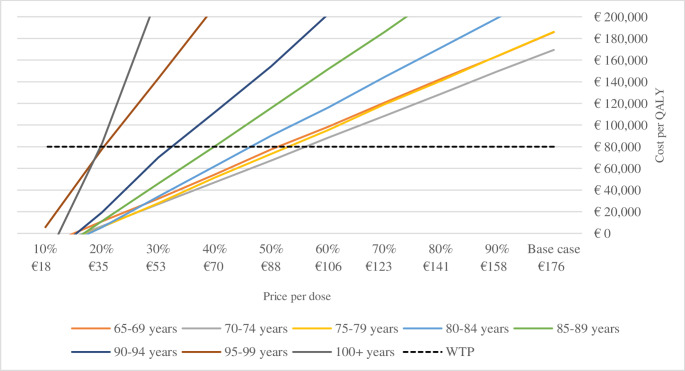
Cost per QALY at different prices of the vaccine for scenario (II) using observational data

At the current RZV market price in Sweden of 176 Euro per dose, the cost per QALY averaged 147 110 Euro for individuals 65–69 years old in scenario (I) with higher vaccine efficacy, while lower vaccine effectiveness depicted in scenario (II) resulted in an average cost per QALY of 185 783 Euro. The lowest cost per QALY was seen for the age group 70–74 years-old, estimated at 133 367 and 169 391 Euro for scenario (I) and (II) respectively. The results are depicted in Figs. 2 and 3. These results are expected, as the age-group 70-74 have a higher risk of HZ and PHN than the younger age-group, while accumulating more years of the preventive positive effect of the vaccine compared to the older age-cohorts.

For the RZV to be deemed cost-effective in scenario (I) and (II) at a WTP of 80,000 Euros per QALY gained, the price of the RZV would have to drop to 80–105 Euro per dose, i.e., 50–60% of the basecase price. These results are depicted in Figs. 2 and 3. In Online Resource 1, results are shown for the combined 65+, 70 + and 75 + year old cohorts.

### Sensitivity analyses

**Fig. 4:**
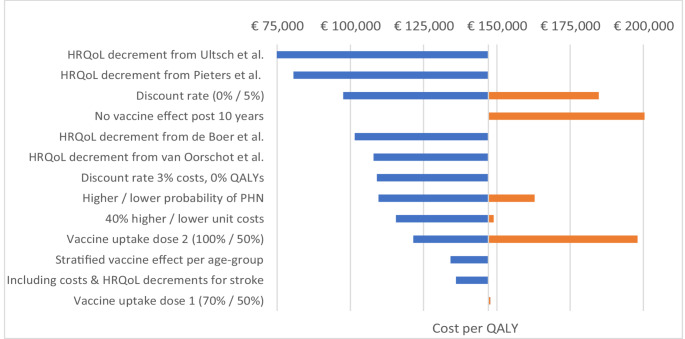
Tornado diagram depicting the cost per QALY for 65–69-year-olds, in relation to the base case results for scenario (I), varying different parameters one-by-one

Several one-way sensitivity analyses were performed, with results presented in Fig. 4; Table 5. In general, varying assumptions did not change the overall conclusion of the base case analysis (both scenario I and II), being that at the current market price of 176 Euro per dose, RZV is not considered cost-effective in relation to no vaccination. On the other hand, what the sensitivity analyses does show is the impact of certain parameters on the price at which the vaccine could be considered cost-effective. The largest impact on these results were generated by using HRQoL decrements from Ultsch et al. [44] or Pieters et al. [16], with larger HRQoL decrements related to HZ and PHN than assumed in the base case analysis. In relation to the base case results in scenario (I) with clinical trial data, using the HRQoL decrements by Ultsch et al. rendered the RZV cost-effective at a WTP of 80,000 Euro per QALY at the current market price of 176 Euro per dose. On the other hand, not extrapolating the vaccine efficacy beyond the 10-year follow-up resulted in an ICER of about 330 000 Euro. In scenario II, using observational data, the ICER ranged between 84 100 to 412 000 Euro per QALY at the current market price. Thus, these parameter uncertainties had a larger impact on the price at which the RZV would be considered cost-effective, than the differences in assumed vaccine efficacy in the two scenarios.

**Table 5:** Results from one-way sensitivity analyses for 65–69-year-olds in scenario (I) using clinical trials data and scenario (II) using observational data

Analysis	ICER Scenario (I) Euro (€)	ICER scenario (II) Euro (€)
Basecase 65–69 year olds	147 110	185 783
0% discount on costs and effects 5% discount on costs and effects	97 574 184 794	124 829 229 432
Vaccine uptake dose 1:		
1. 50% 2. 70%	1. 183 840 2. 146 407	1. 229 690 2. 185 757
Vaccine uptake dose 2:		
1. 50% 2. 100%	1. 147 261 2. 119 893	1. 245 100 2. 155 228
No protection from vaccination after 10 years	330 442	411 719
Including costs and HRQoL decrements due to the increased risk of stroke	136 040	189 962
Vaccine effect stratified by age-group (see Online resource 1)	134 189	188 096
Same health state values for HZ and PHN as reported in the following studies (see Online resource 1):		
1. Pieters et al. [12] 2. van Oorschot et al. [13]/Curran et al. [14] 3. Ultsch et al. [15] 4. de Boer et al. [16]	1. 80 607 2. 107 943 3. 59 301 4. 101 483	1. 114 429 2. 153 431 3. 84 119 4. 143 236
Proportion of PHN:		
1. Lower estimate 2. Higher estimate	1. 162 920 2. 109 602	1. 230 362 2. 154 842
Unit costs:		
1. 40% lower than basecase 2. 40% higher than basecase	1. 148 890 2. 115 581	1. 202 678 2. 171 069

## Discussion

This paper examines the cost-effectiveness of a two-dose vaccination against HZ using the RZV vaccine compared to no vaccination from a health care perspective in Sweden. The cost of the vaccine is put in relation to potentially avoided costs based on reduced healthcare utilization and medication needs, as well as improved quality of life due to the efficacy of RZV to prevent HZ and its complications. As the literature has shown varying degrees of protection from the vaccination, two different scenarios have been applied, where in scenario (I) the vaccine efficacy data comes from clinical trials, and in scenario (II) the vaccine effectiveness data is derived from observational studies. The basecase results indicate that RZV cannot be considered cost-effective in Sweden at a price per dose of 176 Euro, regardless of which vaccine efficacy estimate that is used. If the price per dose were to decrease by 40–50%, RZV could be considered cost-effective at a willingness-to-pay threshold of 80,000 Euro per QALY gained for individuals in the age-cohort 65–69. The vaccine is more cost-effective in the age-cohort 70–74, due to the higher probability of HZ and PHN (compared to 65–69-year-olds) and thus greater savings and HRQoL gains. The populations that are 80 + year old will also not accumulate as many cases of HZ and PHN as the younger cohorts, generating fewer additional QALYs and less cost-offsets. Thus, in the case of a vaccine price reduction, a suitable target-age for vaccination is most likely the 70–74 year-old age group, in light of the health economic returns.

A critical review of published cost-effectiveness analyses of the RZV concluded that out of 18 studies, 15 showed cost-effectiveness, albeit at varying cost of the vaccine [15]. In a study from Belgium with the same time- and health care perspective, cost-effectiveness was investigated for an immunocompetent group, and showed that at a vaccine price per dose of 55 Euros (market price 176 Euro in Sweden), the RZV was cost-effective at a WTP threshold of 40,000 Euro per QALY gained for individuals aged 50–85 years [16]. A German cost-effectiveness analysis reported an even lower ICER of 24,000 Euro at a vaccine price of 84 Euro [45]. A Dutch study with a time horizon of 15 years and a societal perspective concluded that at a general WTP threshold of 20,000 Euro per QALY gained, the vaccine price needed to be 109 Euro for 70-year-olds and 64 Euro for 50-year-olds for RZV to be considered cost-effective [17]. However, assumptions regarding waning of vaccine protection, HRQoL decrements related to HZ and PHN, time perspective and other parameters differ, making direct comparisons difficult. In addition, as pointed out by Bilcke et al. [46], sponsorship bias is a significant issue in cost-effectiveness analyses of the HZ vaccine. Industry-funded economic evaluations have used more favorable parameters, resulting in estimates indicating better value for money of the HZ vaccine.

What is important on the other hand, is how these differences in results may guide decisions in vastly different directions. While the budgetary impact of funding the vaccine publicly is extensive and parameter uncertainty largely impacts on whether the RZV is deemed cost-effective or not, it may hamper equitable access to care between countries. The RZV was recommended for individuals at various starting ages (between 50–65 years and older) within national vaccination programs in the US in 2017, Canada in 2018, Germany in 2019, Spain in 2021 and the UK in 2023 [47]. However, considering the apparent impact of parameter uncertainty, it may guide decision-makers in other countries in different directions. Sensitivity analyses in this study did not change the base case conclusion that the vaccine is not cost-effective at the current market price. However, the analyses revealed that changing certain parameters had a large impact on the price at which the RZV would be deemed cost-effective. For instance, applying HRQoL decrements for HZ and PHN previously used in European cost-effectiveness analyses of the RZV generated ICERs ranging between 59 301–107 943 Euro using clinical trials data [16–18, 44]. Using observational data and assuming a vaccine uptake of 50% of the second dose would yield an ICER of roughly 250,000 Euro per QALY gained. Public procurement at a WTP of 80,000 Euro in Sweden per QALY gained would thus be recommended at the current market price, using the most optimal parameter inputs. If instead other equally likely assumptions were made, it would lead to recommendations of procurement at less than 10% of the current market price.

After conducting our analyses, the Swedish Public Health Agency conducted and published a report on the cost-effectiveness analysis of the RZV [48]. By employing a societal perspective, using other sources and thus some other estimates for input parameters, including some differences in terms of assumptions, such as assuming full coverage of the second vaccine dose, the overall conclusion does not differ from this analysis. The Public Health Agency found that the RZV is not cost-effective at the current market price, and sees a need for a price drop of approximately 30% for the 65-year-old age-cohort for it to be deemed cost-effective. Their analyses showed that indirect costs amounted to 29% of total cost-offsets, which may be one reason as to why the ICER was higher in our basecase analyses [48].

What may additionally impact cost-effectiveness results and thus vaccination programme recommendations are future observational studies. The discrepancy in vaccine efficacy between the clinical trials and the observational studies could be explained by many factors, e.g. different populations (clinical trials excluding immunocompromised individuals for whom the vaccine has been seen as less effective [49]) and different definition of HZ cases, where observational studies rely on diagnosis codes or prescription claims. This means that the true number of cases may be underrepresented if not all cases seek healthcare. With more observational data accumulating with the introduction of the RZV, future observational studies may impact on cost-effectiveness conclusions. Furthermore, just like HZ has been associated with an increased risk of stroke, there are emerging evidence on HZ being a risk factor for other conditions, such as dementia. A protective effect of the vaccine against further conditions would likely impact the cost-effectiveness [50].

This study is the first health economic evaluation of the RZV which evaluates the cost-effectiveness using vaccine efficacy data from the 10-year follow-up of the original clinical trial and comparing these results to real-world observational data of vaccine effectiveness. A further strength is that we used individual level data regarding the incidence of HZ and healthcare utilization, and that all assumptions were made in consultation with experts in the field. The model structure used is inspired by and closely resembles other model structures used in economic evaluations of HZ. The model’s ability to predict the number of individuals developing HZ has been validated by comparing it with data on the annual incidence of HZ among individuals in Sweden. Experts have been involved throughout the process and have validated the input data for the model. The model itself has also been validated by several health economists with knowledge of Markov modeling. Further, parameter uncertainty in economic models of the RZV, which have been highlighted in critical reviews, have been addressed in this paper to examine its effect on the results.

However, certain assumptions have not been varied in sensitivity analyses. These include costs specific for patients developing PHN, assuming the entire cohort is vaccinated in the first year, or the dosages and duration of medication prescribed. Sensitivity analyses for these assumptions have not been performed due to either a lack of reliable information or because variations in these assumptions were considered to have a minor impact on the results. In addition, we did not assume any side effects of the vaccine, which may both impact on HRQoL as well as costs. Neither did we assume a different HRQoL decrement from breakthrough cases of HZ following vaccination, which previously has been reported [51]. Indirect costs were excluded due to the age of the population and because analyses were initially made for decision-makers whom primarily were interested in a healthcare perspective.

Individuals who develop HZ have an increased risk of stroke, which is why costs and quality of life impacts from stroke have been included in a sensitivity analysis, something that previous models on the cost-effectiveness of the RZV have failed to include. As stroke also impacts mortality and give rise to complications persisting for more than one year, a more complex model would have been required to include this effect in the basecase analyes. However, adding this complexity was not deemed to have had any major impact on the results. If mortality and other potential risks associated with HZ had been included, it is possible that the results in the model are slightly underestimated, meaning that the cost per QALY for the RZV could have been slightly lower.

In presenting the results, we have considered a WTP of 80,000 Euro per QALY. The choice of willingness-to-pay is based on historical data on decisions for reimbursement of drugs in Sweden. However, there is currently no established WTP threshold for health care interventions in Sweden.

## Conclusion

The price per dose of the RZV for preventing herpes zoster for the age-cohort 65–69 years old would need to drop to 80–105 Euros (drop by 40–50% of the current market price) for it to be considered cost-effective within the Swedish context given certain parameter choices. The recommended price range for cost-effectiveness is dependent on which efficacy data is used to economically evaluate the vaccine – clinical trials or observational data. Parameter uncertainty, such as health state values, has a great impact on the results and using other assumptions in the basecase analysis would yield significantly different procurement and policy recommendations regarding the price.

## Supplementary Information

Below is the link to the electronic supplementary material.

Supplementary Material 1

## Data Availability

Decision-analytic model is available upon request

## Declarations

### Data

Data available upon ethical approval.
