# Supplementary material for: Informing decisions in light of parameter uncertainty – an economic evaluation of the adjuvanted recombinant herpes zoster vaccine in Sweden
Source: Eur J Health Econ. 2025 Oct 14;27(3):637–49. doi: 10.1007/s10198-025-01829-9 (PMC13190775; doi:10.1007/s10198-025-01829-9)
Supplement: Supplementary file 1 — Supplementary Material 1 [file 10198_2025_1829_MOESM1_ESM.docx]

Informing decisions in light of parameter uncertainty – an economic evaluation of the adjuvanted recombinant herpes zoster vaccine in Sweden

**Journal: European Journal of Health Economics**

**Authors**:

Camilla Nystrand^1,2^

Katarina Widgren^3,5^

Shuang Hao^2,4^

Emelie Heintz^1,2^

Vibeke Sparring^1,2^

**Affiliations**:
^1^ Department of Learning, Informatics, Management and Ethics, Karolinska Institute, Stockholm, Sweden
^2^ Center for Health Economics, Informatics and Healthcare Research, Health Care Services Stockholm County (SLSO), Region Stockholm, Sweden
^3^ Department of Communicable Disease Control and Prevention, Region Stockholm, Stockholm, Sweden
^4^ Department of Medical Epidemiology and Biostatistics, Karolinska Institute, Stockholm, Sweden
^5^ Department of Medicine, Solna, Karolinska Institute, Stockholm, Sweden

Correspondence 1^st^ author: Camilla.nystrand-lansman@regionstockholm.se

Appendix to manuscript

Informing decisions in the light of parameter uncertainty – an economic evaluation of the adjuvanted recombinant herpes zoster vaccine in Sweden for the population of 65 years and above

[Methods 3](#_Toc168042448)

[Meta-analysis of the probability of developing postherpetic neuralgia (PHN) 3](#_Toc168042449)

[Stroke related inputs 4](#_Toc168042450)

[Health-related quality of life for HZ and PHN 5](#_Toc168042451)

[The efficacy of RZV 5](#_Toc168042452)

[Evidence from clinical studies 5](#_Toc168042453)

[Evidence from observational studies 5](#_Toc168042454)

[Age-specific vaccine efficacy 6](#_Toc168042455)

[Results 7](#_Toc168042456)

[Cost per QALY at varying prices of the vaccine 7](#_Toc168042457)

[Sub-group analysis: cost-effectiveness by gender 7](#_Toc168042458)

[References 8](#_Toc168042459)

# Methods

## Meta-analysis of the probability of developing postherpetic neuralgia (PHN)

To investigate the occurrence of postherpetic neuralgia (PHN), a meta-analysis of results from European studies was conducted. In an HTA report from Belgium in the year 2022, a literature search regarding the probability of developing PHN given HZ was performed [1]. The HTA report included two systematic reviews and selected European studies from these reviews based on the following criteria: studies with less than 10% attrition, definition of PHN as pain persisting for more than 90 days after the first symptoms of HZ, a study population size of at least 400 HZ patients (where at least 15% had PHN to allow for age-stratified estimates) and published after the year 2000. Eight studies met all criteria. An additional search was conducted in the HTA-report to include recently published articles, resulting in an additional 12 studies relevant to the research question. These 12 studies were used in a meta-analysis conducted by the authors to estimate the average prevalence of PHN in different age groups.

Data regarding the number of cases of HZ and PHN within relevant age groups (65-69 years, 70-74 years, 75-79 years, 80-84 years, and 85 years and older) were extracted from the 12 studies. Subsequently, separate meta-analyses were conducted for each age group. A random-effects analysis was used for all meta-analyses to account for variance between and within the studies. The data used for the meta-analyses are presented in Table S1 below.

**Table S1**. Data extraction of number of HZ and PHN cases used in the meta-analyses.

| **Author and year** | **Number of HZ cases** | **Number of PHN cases** | | | | |
| --- | --- | --- | --- | --- | --- | --- |
|  | **Age group** | | | | | |
|  | **65+** | **65-69** | **70-74** | **75-79** | **80-84** | **85+** |
| Mick 2010 [2] | 777 | 223 | 290 | 290 | 315 | 315 |
| Duracinsky 2015 [3] | - | - | 61 | 71 | 54 | 88 |
| Hillebrand 2015 [4] | 215 959 | 37 793 | 43 192 | 43 192 | 44 056 | 44 056 |
| Schröder 2017 [5] | 442 979 | 119 604 | 119 604 | 119 604 | 119 604 | 119 604 |
| Ultsch 2013 [6] | 5 384 | 264 | 420 | 420 | 420 | 420 |
| Schmidt-Ott 2018 [7] | 513 | 76 | 67 | 46 | 73 | 73 |
| Helgason 2000 [8] | 421 | 58 | 118 | 118 | - | - |
| Gialloreti 2010 [9] | 5675 | 301 | 573 | 573 | 664 | 664 |
| Bricout 2014 [10] | 413 | 78 | 117 | 117 | 117 | 117 |
| Alicino 2017 [11] | 598 | 89 | 68 | 91 | 86 | 171 |
| Opstelten 2002 [12] | 837 | 28 | 28 | 75 | 75 | 75 |
| Opstelten 2007 [13] | 598 | 46 | 46 | 46 | 46 | 46 |
| Muñoz-Quiles 2018 [14] | 87 086 | 12 192 | 16 633 | 16 633 | 18 549 | 18 549 |
| Sundström 2015 [15] | 13 296 | 904 | 1 223 | 1 290 | 1 502 | 1 502 |
| Gauthier 2009 [16] | 25 002 | 3 250 | 3 750 | 4 500 | 4 750 | 4 750 |
| Yanni 2018 [17] | 18 583 | 1 654 | 2 323 | 2 323 | 2 304 | 2 304 |
| Amirthalingam 2018 [18] | 26 257 | 3 623 | 3 623 | 3 623 | 3 623 | 3 623 |
| Forbes 2016 [19] | 119 413 | 4 299 | 13 016 | 13 016 | 15 285 | 15 285 |
| Walker 2018 [20] | - | - | - | 368 | 368 | 368 |

In Table S2 below, the results from the meta-analyses are presented. The probability of developing PHN for HZ patients varies between 12% for the youngest and 16% for the oldest age-group.

**Table S2.** The probability of developing PHN given HZ, divided by age group.

| **Age grop** | **Probability (95% CI)** |
| --- | --- |
| 65-69 | 11.17 (7.7-16.5) |
| 70-74 | 15 (12-18.3) |
| 75-79 | 15 (12-18.3) |
| 80-84 | 15.1 (12.3-18.2) |
| 85+ | 16.2 (13.3-19.3) |

## Stroke related inputs

Individuals who develop HZ are at an increased risk of stroke. In a meta-analysis from 2017, the elevated risk of stroke was examined, and results were presented for the first year after HZ [21]. Given that stroke results in reduced quality of life and an increased need for care, thereby affecting healthcare costs, the increased risk of stroke has been considered in a sensitivity analysis. In this sensitivity analysis, the model accounts for individuals experiencing diminished quality of life and increased care needs due to both HZ and stroke. No impact on mortality has been considered in this analysis.

The tables below present the data used in the model for the sensitivity analysis, where stroke is included as a complication.

**Table S3.** Increased risk of stroke, given HZ

| **Marra 2017 [21]** | **Relative risk (95% CI)** |
| --- | --- |
| 1 month post HZ | 1.78 (1.7-1.88) |
| 3 months post HZ | 1.43 (1.38-1.47) |
| 1 year post HZ | 1.19 (1.13-1.24) |

The average risk of stroke at any time during the first year after HZ has been calculated by applying the "area under the curve" method, which considers the time interval for which the risk is measured. The average risk used in the model is therefore 1.4 during the first year.

The increased risk of experiencing a stroke after a HZ infection was applied to the baseline risk of stroke (incidence), which is on average 0.3 percent for individuals aged 65 and older, estimated from the Swedish National Board of Health and Welfare's open statistical database [22].

The health-related quality of life for individuals after a stroke has been estimated in a meta-analysis from 2022 [23]. The table below presents the average quality of life based on the study's findings for the first year.

**Table S4.** Health related quality of life after stroke

| **Joundi 2022 (49)** | **Health related quality of life (95% CI)** |
| --- | --- |
| 1 month past stroke | 0.65 (0.63-0.67) |
| 1 year past stroke | 0.65 (0.59-0.71) |

The average quality of life for individuals who have experienced a stroke at any time during the first year after the stroke has been calculated using the "area under the curve" method. The average quality of life used in the model is therefore 0.65.

Stroke patients require both primary care, specialized outpatient care, and inpatient care. In a Swedish study from 2017, the costs for stroke patients were estimated [24]. The costs were estimated for the year 2016, and in the table below, the costs are adjusted to the year 2021, which is the same year used for other costs in the model.

**Table S5.** Costs related to stroke in Sweden (Euro, 2021)

|  | **Primary care** | **Specialised outpatient care** | **Inpatient care** |
| --- | --- | --- | --- |
| Hemorrhagic stroke | 897 | 1 922 | 25 622 |
| Ischemic stroke | 1 189 | 1 983 | 16 442 |

## Health-related quality of life for HZ and PHN

In sensitivity analysis, utility decrements were derived from other health economic evaluations of the RZV conducted in a European setting. The studies were selected and data extracted from a critical review of economic evaluations of the RZV [25]. The utility decrements used for HZ and PHN in the sensitivity analyses are presented in the table below.

**Table S6.** Utility decrements related to herpes zoster (HZ) and post herpetic neuralgia (PHN) used in sensitivity analyses.

| **Study** | **HZ** | **PHN** |
| --- | --- | --- |
| **Pieters [26]** | **65-69**: 0.00589 **70+**: 0.01173 | **65-69**: 0.20439  **70+**: 0.26133 |
| **Oorschot [27] / Curran [28]** | **65-69**: 0.018  **70+**: 0.019 | **65+**: 0.158 |
| **De Boer [29]** | **65+**: 0.057 | **65+**: 0.057 |
| **Ultsch [30]** | **65-69**: 0.033  **70+**: 0.031 | **65-69**: 0.263  **70+**: 0.296 |

## The efficacy of RZV

### Evidence from clinical studies

Vaccine efficacy has been estimated in an interim follow-up study of 10 years [31]. The table below shows the effects during these years. On average, the vaccine efficacy waned by 3.1% annually.

**Table S7.** Vaccine efficacy in clinical trials and long-term follow-up of clinical trials

| **Year** | **Vaccine efficacy (95% CI), %** |
| --- | --- |
| 1 | 97.7 (93.1-99.5) |
| 2 | 92.7 (86.2-96.6) |
| 3 | 92.4 (85.0-96.6) |
| 4 | 89.8 (80.3-95.2) |
| Gap between the clinical trials and the long-term follow-up | |
| 6 | 88.5 (74.9-95.6) |
| 7 | 83.3 (67.2-92.4) |
| 8 | 84.2 (67.9-93.1) |
| 9 | 72.7 (51.0-85.7) |
| 10 | 73.2 (46.9-87.6) |

It was therefore assumed in the basecase analysis that post-ten years; the vaccine would wane further by the same annual rate. In a sensitivity analyses, other assumptions were made.

### Evidence from observational studies

Three observational studies have been published that examined the vaccine effectiveness of RZV for individuals (regardless of medical history) or for non-immunocompromised individuals (as in the clinical studies investigating vaccine efficacy). Two of these studies assessed vaccine efficacy after an average of seven months [32, 33], while the third study examined vaccine efficacy after an average of two years [34]. Due to challenges in combining results from studies with different follow-up intervals, the third study was excluded. Results from a random-effects meta-analysis was retrieved [35]. The measurement of vaccine effectiveness at seven months was then assumed to represent the vaccine effectiveness during the first year.

The results from the meta-analysis showed a vaccine efficacy of 79.2% (95% CI 57.6-89.7%). In the following years, a waning of 3.1% was assumed, based on data from the clinical studies.

The waning over time is shown in Figure S1 below.

**Figure S1.** Effect waning over time

## Age-specific vaccine efficacy

From the clinical study of vaccine efficacy, results for the first 3 years were available for the age-groups 60-69, 70-79 and 80+ year olds. For observational data, age-specific vaccine effectiveness was available for the same age-cohorts for the first year. The age-specific estimates are shown in the table below. In a sensitivity analyses, these estimates were used, and the same waning rate of 3.1% as used in the basecase model were applied in the following years.

**Table S8.** Age-specific vaccine efficacy during the first 3 years (scenario I) and first year (scenario II) post vaccination with 2 doses of the RZV

| **Clinical data (scenario I)** | **Mean (95% CI) vaccine efficacy first 3 years** | **Source** |
| --- | --- | --- |
| 60–69-year-old | 0.974 (0.901-0.997) | [36] |
| 70–79-year-old | 0.913 (0.86-0.949) | [37] |
| 80+ year old | 0.914 (0.802-0.970) | [37] |
|  |  |  |
| **Observational data (scenario II)** | **Mean (95% CI) vaccine effectiveness first year** | **Source** |
| 60–69-year-old | 0.786 (0.635-0.972) | Own meta-analysis, see chapter “Evidence from observational studies” |
| 70–79-year-old | 0.781 (0.640-0.953) |  |
| 80+ year old | 0.741 (0.634-0.866) |  |

# Results

## Cost-effectiveness per age-group

Results below are shown for each age cohort between 70-89 years old. The results for the age-group 65-69 is shown in the main manuscript, and the probability of cost-effectiveness for the even older age-groups is zero percent for a willingness-to-pay of 2 million SEK (roughly 197,000 Euro), hence not shown in this Online Resource.

**Table S9.** Number of cases, discounted QALYs, discounted costs and incremental cost-effectiveness (2021 Euro) related to the RZV (using efficacy data from clinical or observational studies) vs. no vaccination for individuals aged 70-74 years. Mean estimates and 95% uncertainty intervals (UI) are shown

| **Outcomes** | **RZV** | **No vaccination** | | **Incremental** | |
| --- | --- | --- | --- | --- | --- |
| Scenario (I) – efficacy data from clinical studies | | | | | |
| HZ cases | 47 830 | 72 811 | | -24 981 | |
| PHN cases | 8 749 | 13 267 | | -4 518 | |
| QALYs | 4 252 032 | 4 251 414 | | 619 (564 – 676) | |
| Vaccination costs | 108 426 625 | |  | |  |
| Direct costs due to HZ and complications | 527 529 626 | 553 659 012 | | -26 129 386 (-21 942 793 – -32 227 450) | |
| Net cost |  |  | | 82 297 239 (76 199 175 – 86 483 886) | |
| Incremental cost/QALY |  |  | | 133 367 | |
| Scenario (II) – efficacy data from observational studies | | | | | |
| HZ cases | 52 044 | 72 808 | | -20 764 | |
| PHN cases | 9 514 | 13 270 | | -3 756 | |
| QALYs | 4 251 803 | 4 251 290 | | 513 (452 – 573) | |
| Vaccination costs | 108 426 625 |  | |  | |
| Direct costs due to HZ and complications | 532 275 393 | 554 140 816 | | -21 865 423 (-17 795 548 – -27 021 475) | |
| Net cost |  |  | | 86 561 202 (81 405 150 – 90 631 077) | |
| Incremental cost/QALY |  |  | | 169 391 | |

**Table S10.** Number of cases, discounted QALYs, discounted costs and incremental cost-effectiveness (2021 Euro) related to the RZV (using efficacy data from clinical or observational studies) vs. no vaccination for individuals aged 75-79 years. Mean estimates and 95% uncertainty intervals (UI) are shown.

| **Outcomes** | **RZV** | **No vaccination** | | **Incremental** | |
| --- | --- | --- | --- | --- | --- |
| Scenario (I) – efficacy data from clinical studies | | | | | |
| HZ cases | 33 911 | 53 804 | | -19 893 | |
| PHN cases | 6 270 | 9 910 | | -3 640 | |
| QALYs | 2 970 784 | 2 970 279 | | 506 (457 – 553) | |
| Vaccination costs | 97 642 937 | |  | |  |
| Direct costs due to HZ and complications | 387 284 680 | 411 750 234 | | -24 465 553 (-20 045 718 – -30 770 627) | |
| Net cost |  |  | | 73 177 384 (66 872 310 – 77 597 219) | |
| Incremental cost/QALY |  |  | | 145 120 | |
| Scenario (II) – efficacy data from observational studies | | | | | |
| HZ cases | 37 368 | 53 796 | | -16 428 | |
| PHN cases | 6 909 | 9 918 | | -3 008 | |
| QALYs | 2 970 660 | 2 970 242 | | 418 (364 – 472) | |
| Vaccination costs | 97 642 937 |  | |  | |
| Direct costs due to HZ and complications | 391 542 489 | 411 863 792 | | -20 321 303 (-16 460 464 – -25 622 012) | |
| Net cost |  |  | | 77 321 634 (72 020 925 – 81 182 473) | |
| Incremental cost/QALY^c^ |  |  | | 186 079 | |

**Table S11.** Number of cases, discounted QALYs, discounted costs and incremental cost-effectiveness (2021 Euro) related to the RZV (using efficacy data from clinical or observational studies) vs. no vaccination for individuals aged 80-84 years. Mean estimates and 95% uncertainty intervals (UI) are shown.

| **Outcomes** | **RZV** | **No vaccination** | | **Incremental** | |
| --- | --- | --- | --- | --- | --- |
| Scenario (I) – efficacy data from clinical studies | | | | | |
| HZ cases | 14 867 | 24 576 | | -9 708 | |
| PHN cases | 2 803 | 4 620 | | -1 817 | |
| QALYs | 1 292 915 | 1 292 664 | | 251 (230 – 274) | |
| Vaccination costs | 58 855 435 | |  | |  |
| Direct costs due to HZ and complications | 179 234 732 | 194 007 270 | | -14 772 538 (-11 794 680 – -18 853 066) | |
| Net cost |  |  | | 44 082 898 (40 002 370 – 47 060 756) | |
| Incremental cost/QALY |  |  | | 175 991 | |
| Scenario (II) – efficacy data from observational studies | | | | | |
| HZ cases | 16 581 | 24 572 | | -7 990 | |
| PHN cases | 3 128 | 4 623 | | -1 496 | |
| QALYs | 1 292 893 | 1 292 686 | | 207 (181 – 232) | |
| Vaccination costs | 58 855 435 |  | |  | |
| Direct costs due to HZ and complications | 181 857 017 | 194 063 469 | | -12 206 452 (-15 842 727 – -9 624 348) | |
| Net cost |  |  | | 46 648 984 (43 012 709 – 49 231 088) | |
| Incremental cost/QALY |  |  | | 226 592 | |

**Table S12.** Number of cases, discounted QALYs, discounted costs and incremental cost-effectiveness (2021 Euro) related to the RZV (using efficacy data from clinical or observational studies) vs. no vaccination for individuals aged 85-89 years. Mean estimates and 95% uncertainty intervals (UI) are shown.

| **Outcomes** | **RZV** | **No vaccination** | | **Incremental** | |
| --- | --- | --- | --- | --- | --- |
| Scenario (I) – efficacy data from clinical studies | | | | | |
| HZ cases | 5 715 | 9 819 | | -4 105 | |
| PHN cases | 1 104 | 1 898 | | -793 | |
| QALYs | 524 342 | 524 229 | | 113 (102 – 125) | |
| Vaccination costs | 33 839 787 | |  | |  |
| Direct costs due to HZ and complications | 74 819 406 | 83 077 785 | | -8 258 379 (-6 475 453 – -10 730 283) | |
| Net cost |  |  | | 25 581 409 (23 109 504 – 27 364 334) | |
| Incremental cost/QALY |  |  | | 226 659 | |
| Scenario (II) – efficacy data from observational studies | | | | | |
| HZ cases | 6 435 | 9 819 | | -3 384 | |
| PHN cases | 1 244 | 1 898 | | -654 | |
| QALYs | 524 386 | 524 292 | | 93 (81 – 105) | |
| Vaccination costs | 33 839 787 |  | |  | |
| Direct costs due to HZ and complications | 76 465 750 | 83 361 661 | | -6 895 912 (-5 301 658 – -9 342 161) | |
| Net cost |  |  | | 26 943 875 (24 497 626 – 28 538 129) | |
| Incremental cost/QALY |  |  | | 290 061 | |

## Cost-effectiveness 65+ year olds

**Table S13**. Number of cases, discounted QALYs, discounted costs and incremental cost-effectiveness (2021 Euro) related to the RZV (using efficacy data from clinical or observational studies) vs. no vaccination for individuals aged 65+ years. Mean estimates and 95% uncertainty intervals (UI) are shown.

| **Outcomes** | **RZV** | **No vaccination** | | **Incremental** | |
| --- | --- | --- | --- | --- | --- |
| Scenario (I) – efficacy data from clinical studies | | | | | |
| HZ cases | 162 649 | 250 028 | | -87 379 | |
| PHN cases | 29 699 | 45 454 | | -15 755 | |
| QALYs | 14 561 967 | 14 559 850 | | 2 117 (1 909 – 2 323) | |
| Vaccination costs | 428 509 216 | |  | |  |
| Direct costs due to HZ and complications | 1 827 475 467 | 1 929 641 378 | | -102 165 912 (-83 393 135 – -128 204 349) | |
| Net cost |  |  | | 326 343 304 (300 304 867 – 345 116 081) | |
| Incremental cost/QALY |  |  | | 163 397 | |
| Scenario (II) – efficacy data from observational studies | | | | | |
| HZ cases | 177 585 | 250 017 | | -74 432 | |
| PHN cases | 32 400 | 45 464 | | -13 064 | |
| QALYs | 14 561 570 | 14 559 817 | | 1 753 (1 535 – 1 973) | |
| Vaccination costs | 428 509 216 |  | |  | |
| Direct costs due to HZ and complications | 1 845 441 302 | 1 930 420 817 | | -84 979 397 (-68 430 637 – -107 582 340) | |
| Net cost |  |  | | 343 529 819 (320 926 876 – 360 078 579) | |
| Incremental cost/QALY |  |  | | 208 376 | |

## Cost-effectiveness acceptability curves for the basecase scenario

**Figure S2**. Probability of cost-effectiveness for scenario (I) using various willingness-to-pay thresholds for the age groups 65-89.

**Figure S3**. Probability of cost-effectiveness for scenario (II) using various willingness-to-pay thresholds for the age groups 65-84.

## Cost per QALY at varying prices of the vaccine for merged age-groups, weighted by the population size in each age group.

**Figure S3**. Cost per QALY in scenario (I) and (II) for different prices for the vaccine, for 65+, 70+ and 75+ year-olds

## Sub-group analysis: cost-effectiveness by gender

Table S14 and S15 below shows the basecase results for females and males separately.

**Table S14**. Results from sub-group analyses for 65+ year olds - females only (2021 Euro)

| **Outcomes** | **RZV** | **No vaccination** | | **Incremental** | |
| --- | --- | --- | --- | --- | --- |
| Scenario (I) – efficacy data from clinical studies | | | | | |
| HZ cases | 102 263 | 156 554 | | -54 291 | |
| PHN cases | 18 713 | 28 523 | | -9 810 | |
| QALYs | 7 377 149 | 7 375 940 | | 1 210 (1 089 – 1 336) | |
| Vaccination costs | 228 319 514 | |  | |  |
| Direct costs due to HZ and complications | 1 024 934 182 | 1 090 730 156 | | -65 795 974 (-83 188 206 – -52 974 877) | |
| Net cost |  |  | | 162 523 540 (145 131 308 – 175 344 637) | |
| Incremental cost/QALY |  |  | | 145 193 | |
| Scenario (II) – efficacy data from observational studies | | | | | |
| HZ cases | 111 495 | 156 533 | | -45 037 | |
| PHN cases | 20 397 | 28 544 | | -8 147 | |
| QALYs | 7 376 927 | 7 375 925 | | 1 002 (881 – 1 124) | |
| Vaccination costs | 228 319 514 |  | |  | |
| Direct costs due to HZ and complications | 1 036 139 197 | 1 090 696 999 | | -54 557 802 (-69 460 738 – -43 523 895) | |
| Net cost |  |  | | 173 761 712 (158 858 776 – 184 795 776) | |
| Incremental cost/QALY |  |  | | 188 031 | |

**Table S15.** Results from sub-group analyses for 65+ year olds - males only (2021 Euro)

| **Outcomes** | **RZV** | **No vaccination** | | **Incremental** | |
| --- | --- | --- | --- | --- | --- |
| Scenario (I) – efficacy data from clinical studies | | | | | |
| HZ cases | 58 071 | 90 116 | | -32 045 | |
| PHN cases | 10 541 | 16 286 | | -5 745 | |
| QALYs | 7 041 154 | 7 040 311 | | 844 (764 – 930) | |
| Vaccination costs | 200 189 702 | |  | |  |
| Direct costs due to HZ and complications | 796 867 964 | 832 211 582 | | -35 343 618 (-43 395 736 – -29 485 894) | |
| Net cost |  |  | | 164 846 083 (156 793 965 – 170 703 808) | |
| Incremental cost/QALY |  |  | | 241 087 | |
| Scenario (II) – efficacy data from observational studies | | | | | |
| HZ cases | 63 567 | 90 124 | | - 26 557 | |
| PHN cases | 11 527 | 16 290 | | -4 763 | |
| QALYs | 7 042 018 | 7 041 321 | | 698 (612 – 786) | |
| Vaccination costs | 200 189 702 |  | |  | |
| Direct costs due to HZ and complications | 802 748 522 | 831 926 872 | | -29 178 350 (-36 262 080 – -24 168 427) | |
| Net cost |  |  | | 171 011 352 (163 927 622 – 176 021 275) | |
| Incremental cost/QALY |  |  | | 301 872 | |

# References

1. Roberfroid Dominique, Z.R., Maertens de Noordhout Charline, Thiry Nancy, *Evaluation of shingrix vaccine against herpes zoster*, in *KCE Reports 360*, B.H.C.K.C. (KCE), Editor. 2022, Belgian Health Care Knowledge Centre (KCE): Brussels.

2. Mick, G., et al., *[Burden of herpes zoster and postherpetic neuralgia: Incidence, proportion, and associated costs in the French population aged 50 or over].* Rev Epidemiol Sante Publique, 2010. **58**(6): p. 393-401.

3. Duracinsky, M., et al., *ARIZONA study: is the risk of post-herpetic neuralgia and its burden increased in the most elderly patients?* BMC Infect Dis, 2014. **14**: p. 529.

4. Hillebrand, K., et al., *Incidence of herpes zoster and its complications in Germany, 2005-2009.* J Infect, 2015. **70**(2): p. 178-86.

5. Schröder, C., et al., *Incidence of herpes zoster amongst adults varies by severity of immunosuppression.* J Infect, 2017. **75**(3): p. 207-215.

6. Ultsch, B., et al., *Epidemiology and cost of herpes zoster and postherpetic neuralgia in Germany.* Eur J Health Econ, 2013. **14**(6): p. 1015-26.

7. Schmidt-Ott, R., et al., *Incidence and costs of herpes zoster and postherpetic neuralgia in German adults aged ≥50 years: A prospective study.* J Infect, 2018. **76**(5): p. 475-482.

8. Helgason, S., et al., *Prevalence of postherpetic neuralgia after a first episode of herpes zoster: prospective study with long term follow up.* Bmj, 2000. **321**(7264): p. 794-6.

9. Gialloreti, L.E., et al., *Epidemiology and economic burden of herpes zoster and post-herpetic neuralgia in Italy: a retrospective, population-based study.* BMC Infect Dis, 2010. **10**: p. 230.

10. Bricout, H., et al., *Burden of herpes zoster-associated chronic pain in Italian patients aged 50 years and over (2009-2010): a GP-based prospective cohort study.* BMC Infect Dis, 2014. **14**: p. 637.

11. Alicino, C., et al., *Incidence of herpes zoster and post-herpetic neuralgia in Italy: Results from a 3-years population-based study.* Hum Vaccin Immunother, 2017. **13**(2): p. 399-404.

12. Opstelten, W., et al., *Herpes zoster and postherpetic neuralgia: incidence and risk indicators using a general practice research database.* Fam Pract, 2002. **19**(5): p. 471-5.

13. Opstelten, W., et al., *Predicting postherpetic neuralgia in elderly primary care patients with herpes zoster: prospective prognostic study.* Pain, 2007. **132 Suppl 1**: p. S52-s59.

14. Muñoz-Quiles, C., et al., *Impact of postherpetic neuralgia: A six year population-based analysis on people aged 50 years or older.* J Infect, 2018. **77**(2): p. 131-136.

15. Sundström, K., et al., *Incidence of herpes zoster and associated events including stroke--a population-based cohort study.* BMC Infect Dis, 2015. **31**(15): p. 488.

16. Gauthier, A., et al., *Epidemiology and cost of herpes zoster and post-herpetic neuralgia in the United Kingdom.* Epidemiol Infect, 2009 **137**(1): p. 38-47.

17. Yanni, E.A., et al., *Burden of herpes zoster in 16 selected immunocompromised populations in England: a cohort study in the Clinical Practice Research Datalink 2000-2012.* BMJ Open, 2018. **8**(6): p. e020528.

18. Amirthalingam, G., et al., *Evaluation of the effect of the herpes zoster vaccination programme 3 years after its introduction in England: a population-based study.* Lancet Public Health, 2018. **3**(2): p. e82-e90.

19. Forbes, H.J., et al., *Quantification of risk factors for postherpetic neuralgia in herpes zoster patients: A cohort study.* Neurology, 2016. **87**(1): p. 94-102.

20. Walker, J.L., et al., *Effectiveness of herpes zoster vaccination in an older United Kingdom population.* Vaccine, 2018. **36**(17): p. 2371-2377.

21. Marra, F., J. Ruckenstein, and K. Richardson, *A meta-analysis of stroke risk following herpes zoster infection.* BMC Infectious Diseases, 2017. **17**(1): p. 198.

22. Swedish National Board of Health and Welfare. *Statistics database for diagnoses*. 2021; Available from: <https://sdb.socialstyrelsen.se/if_par/val.aspx>.

23. Joundi, R.A., et al., *Health State Utility Values in People With Stroke: A Systematic Review and Meta-Analysis.* J Am Heart Assoc, 2022. **11**(13): p. e024296.

24. Lekander, I., et al., *Relationship between functional disability and costs one and two years post stroke.* PLoS One, 2017. **12**(4).

25. Giannelos, N., C. Ng, and D. Curran, *Cost-effectiveness of the recombinant zoster vaccine (RZV) against herpes zoster: An updated critical review.* Human Vaccines & Immunotherapeutics, 2023. **19**(1): p. 2168952.

26. Pieters, Z., et al., *Cost-Effectiveness Analysis of Herpes Zoster Vaccination in 50- to 85-Year-Old Immunocompetent Belgian Cohorts: A Comparison between No Vaccination, the Adjuvanted Subunit Vaccine, and Live-Attenuated Vaccine.* Pharmacoeconomics, 2022. **40**(4): p. 461-476.

27. van Oorschot, D.A.M., et al., *Public health impact model estimating the impact of introducing an adjuvanted recombinant zoster vaccine into the UK universal mass vaccination programme.* BMJ Open, 2019. **9**(5): p. e025553.

28. Curran, D.A.-O., et al., *Long-term efficacy data for the recombinant zoster vaccine: impact on public health and cost effectiveness in Germany.* Human vaccines & immunotherapeutics, 2021. **17**(12): p. 5296-5303.

29. de Boer, P.T., et al., *Cost-effectiveness of vaccination of immunocompetent older adults against herpes zoster in the Netherlands: a comparison between the adjuvanted subunit and live-attenuated vaccines.* (1741-7015 (Electronic)).

30. Ultsch B, W.F., Koch J, Siedler A, *Modellierung von epidemiologischen und gesundheitsökonomischen: Effekten von Impfungen zur Prävention von Herpes zoster*. 2017.

31. Strezova, A., et al., *Long-term Protection Against Herpes Zoster by the Adjuvanted Recombinant Zoster Vaccine: Interim Efficacy, Immunogenicity, and Safety Results up to 10 Years After Initial Vaccination.* Open Forum Infect Dis, 2022. **9**(10).

32. Izurieta, H.S., et al., *Recombinant Zoster Vaccine (Shingrix): Real-World Effectiveness in the First 2 Years Post-Licensure.* Clinical Infectious Diseases, 2021. **73**(6): p. 941-948.

33. Sun, Y., et al., *Effectiveness of the Recombinant Zoster Vaccine in Adults Aged 50 and Older in the United States: A Claims-Based Cohort Study.* Clin Infect Dis, 2021. **73**(6): p. 949-956.

34. Sun, Y., et al., *Effectiveness of the recombinant zoster vaccine among Kaiser Permanente Hawaii enrollees aged 50 and older: A retrospective cohort study.* Vaccine, 2021. **39**(29): p. 3974-3982.

35. Mbinta, J.F., et al., *Post-licensure zoster vaccine effectiveness against herpes zoster and postherpetic neuralgia in older adults: a systematic review and meta-analysis.* The Lancet Healthy Longevity, 2022. **3**(4): p. e263-e275.

36. Lal, H., et al., *Efficacy of an adjuvanted herpes zoster subunit vaccine in older adults.* N Engl J Med, 2015. **372**(22): p. 2087-96.

37. Cunningham, A.L., et al., *Efficacy of the Herpes Zoster Subunit Vaccine in Adults 70 Years of Age or Older.* New England Journal of Medicine, 2016. **375**(11): p. 1019-1032.
